# Draft genome sequences of 11 *Xanthomonas* strains associated with bacterial spot disease in Turkey

**DOI:** 10.1099/acmi.0.000586.v3

**Published:** 2023-06-23

**Authors:** Aastha Subedi, Serhat Kara, Yesim Aysan, Gerald V. Minsavage, Sujan Timilsina, Pamela D. Roberts, Erica M. Goss, Jeffrey B. Jones

**Affiliations:** ^1^​ Department of Plant Pathology, University of Florida, Gainesville, Florida, USA; ^2^​ Alata Horticulture Research Institute, Mersin, Turkey; ^3^​ Department of Plant Protection, Cukurova University, Adana, Turkey; ^4^​ Southwest Florida Research & Education Center, University of Florida, Immokalee, Florida, USA; ^5^​ Emerging Pathogens Institute, University of Florida, Gainesville, Florida, USA

**Keywords:** bacterial-spot, *Xanthomonas*, pepper, tomato, eggplant, whole-genome, Turkey

## Abstract

Bacterial spot is an economically significant disease in tomato and pepper-producing countries globally. We report the whole-genome sequence of 11 *Xanthomonas* strains associated with bacterial spot disease on pepper, tomato and eggplant in the Southeastern Anatolia Region, Turkey. This genomic information can be used as a reference to study the genetic diversity of these species and contribute to illuminating pathogen evolution with respect to host specificity.

## Data Summary

The whole-genome sequence assemblies and raw read data have been deposited in GenBank under BioProject number PRJNA932009 and the accession numbers are listed in Table 1. The accession numbers for type strains *X. euvesicatoria* LMG-27970 and *X. perforans* DSM-18975 are JPYC00000000 and JABFFS000000000 respectively.

## Introduction

Bacterial spot is considered a major disease in many regions worldwide where tomatoes and peppers are grown, causing a significant economic impact to growers. Bacterial spot disease is caused by four *Xanthomonas* spp.: *X. euvesicatoria *, *X. perforans *, *X. vesicatoria,* and *X. hortorum* pv. *gardneri* [1–3]. While the severity of the disease can vary depending on the specific bacterial strain [4], and environmental factors [5], yield losses of up to more than 40 % on peppers and severe effects on the yield and quality of tomatoes have been reported [6, 7]. These bacterial pathogens are capable of being disseminated through the movement of seed, infected transplants, contaminated tools, wind-blown rain and dust particles, and insect vectors among others [3]. The disease has been identified in more than 70 countries across all continents except Antarctica [8]. Pepper and tomatoes represent the two major hosts for these pathogens. Prior to 2010, *X. perforans* was only known to cause bacterial spot of tomato. However, in the last decade, a host range shift in the *X. perforans* population to include pepper as a host has been observed in North America [2, 3, 9, 10]. Surveys of *Xanthomonas* populations in other growing regions are needed to identify host-range shifts. There is currently no genomic information available for *Xanthomonas* on pepper and tomato from Turkey, where both crops are the most widely grown vegetables. In 2021, Turkey reported 3 million tons of pepper and 13 million tons of tomato production and was ranked as third and fourth producer, respectively, in the world (FAOSTAT, 2021). Hence, it is crucial to understand the diseases that can cause millions of dollars in losses impacting the economy of the country.

## Methods

### Sample collection and isolation of bacteria

Symptomatic leaves showing symptoms as small, dark-brown lesions surrounded by yellow halo were collected from three different solanaceous plants: pepper, tomato and eggplant from Southeastern Anatolia Region of Turkey in 2020. A small section from the margin of individual lesions was macerated in 25 µl of sterile tap water using sterilized scalpels and forceps. A loopful of the suspension was streaked on nutrient agar (NA) medium (Laboratories, Detroit, MI) in a quadrant streak pattern, and plates were incubated for 72 h at 28 °C until single colonies were observed. An individual yellow colony was streaked on fresh NA and, growth from the 24 h culture was suspended in cryovials containing 30 % glycerol in nutrient broth and stored at −80 °C for further assays.

### Pathogen identification and pathogenicity test

Overnight fresh cultures grown on NA were transferred to nutrient broth and incubated overnight at 28 °C by placing on a shaker at 250 r.p.m. Genomic DNA was extracted from overnight nutrient broth cultures using the Gram-negative bacterial DNA extraction protocol from the Wizard Genomic DNA Purification Kit (Promega, Madison, WI). Initial species confirmation was conducted using species-specific real-time PCR probes to amplify the *hrcN* gene [11]. As explained by [11], each real-time PCR reaction consisted of 0.2 µl of molecular-grade water, 5 µl of 10×PCR buffer, 6.0 µl of 25 nM MgCl_2_, 0.6 µl of 10 mM dNTP, 3.0 µl of each forward and reverse primer at 2 µM, 1.25 µl of each probe at 1 µM, and 0.2 µl of platinum *Taq* per and 2 µl extracted genomic DNA. A sample with a cycle threshold (Ct) value <35 was considered as positive whereas a sample with a Ct value ≥37 was considered as negative [11]. *X. euvesicatoria* strain E3 and *X. perforans* strain 91–118 were used as controls.

Pepper (*Capsicum annuum L*.) cultivar ‘Early California Wonder’ (ECW), and tomato cultivar ‘Bonny Best’ were infiltrated in the underside of leaves with bacterial suspensions adjusted to 10^8^ c.f.u. ml^−1^ (OD_600_=0.3) from overnight cultures grown on NA. Infiltrated leaf areas were monitored for a hypersensitive response (HR) or susceptible reaction. Three strains, *X. perforans* Xp-91–118 (pathogenic on tomato), *X. perforans* Xp-2010 (pathogenic on pepper) and *X. euvesicatoria* Xe_E3 (pathogenic on both tomato and pepper), were used as controls. Because eggplant has not been reported as a host for *X. perforans *, in-planta bacterial growth was assessed for the Tu_04 strain by infiltrating a bacterial suspension adjusted to 10^5^ c.f.u. ml^−1^ into two cultivars of eggplant (Hybrid and Black beauty). Pathogenicity test was conducted once with each test strain and control strain in single leaf while population growth assay was replicated two times.

### Genome sequencing

For whole-genome sequencing, extracted genomic DNA (as explained above) was sent to Microbial Genome Sequencing Center (MIGS; Pittsburgh, PA, USA). Genomes were sequenced using the Illumina Next-Seq2000 platform, generating 151 bp paired-end reads. Sample libraries were prepared using the Illumina DNA Prep kit and IDT 10 bp UDI indices. Demultiplexing, quality control and adapter trimming was performed with bcl-convert (v3.9.3) – a proprietary Illumina software for the conversion of bcl files to basecalls.

### Genome assembly

Raw reads were assembled using pipelines published in Timilsina *et al*. [12]. Briefly, adapters were eliminated by Trim Galore with default settings to generate clean sequences and pair raw reads [13]. Paired reads were assembled using SPAdes (v.3.10.1) with ‘—careful’ switch [14] and contigs with less than 500 bp and *k*-mer coverage of less than 2.0 were eliminated. Validated reads were aligned against the filtered contigs using default parameters of Bowtie 2 (v. 2.3.3) [15]. The generated SAM files were converted to BAM files using SAMtools [16]. Pilon was used to polish the draft genome assemblies with ‘--frags’ setting [17]. Genome statistics were calculated using a python script (https://github.com/sujan8765/nepgorkhey_python/blob/master/genome_stats.py). Pyani (v.0.2.10) [18] was used to calculate an average nucleotide identity (ANI) among assemblies and to reference genomes of type strains of *X. euvesicatoria* LMG-27970 and *X. perforans* DSM-18975. Genome assemblies were annotated using the Prokaryotic Genome Annotation Pipeline v.6.4 from the National Centre for Biotechnology Information [19].

## Results and discussion

**Fig. 1.**
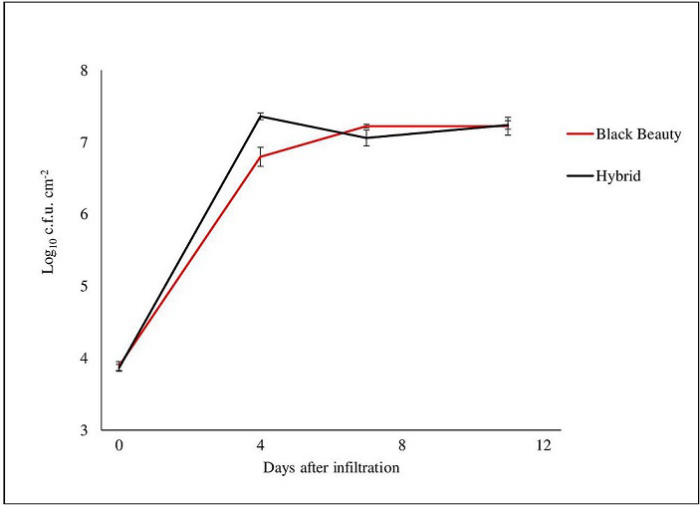
*In planta* bacterial growth of *X. perforans* Tu_04 in two cultivars of eggplant (Black beauty and Hybrid) at various time-points after infiltration with a suspension adjusted to 10^5^ c.f.u. ml^−1^. The error bars indicate standard errors.

**Fig. 2.**
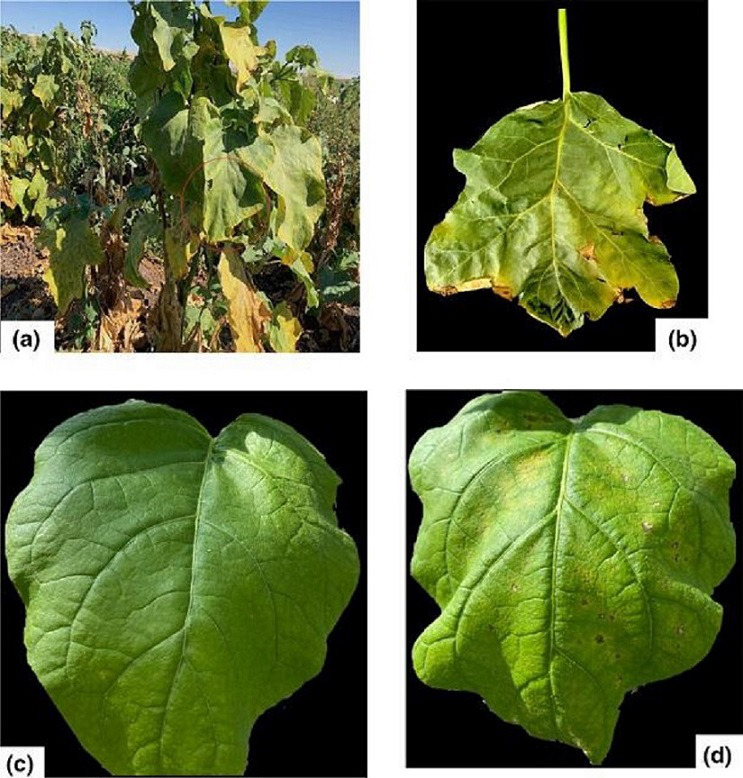
Bacterial spot disease associated with eggplant. (a) Sampled field with symptoms on eggplant. (b) Eggplant leaf with lesion from which isolation was done. (c) Eggplant leaf at day 0 infection. (d) Symptoms developed on artificially inoculated eggplant (cv. Black beauty) in the greenhouse. For the artificial inoculation, the bacterial suspension was adjusted to 10^5^ c.f.u. ml^–1^ and infiltrated into the intercellular spaces of the leaf. The picture was taken at 11 days post-infiltration.

Of 11 strains, five from pepper were identified as *X. euvesicatoria,* and six strains from tomato, pepper and eggplant as *X. perforans* (Table 1). Pathogenicity tests revealed that all strains from tomato and pepper were pathogenic on their host of isolation. *X. perforans* strain Tu_04 showed weak growth in two different eggplant cultivars, reaching populations of only 1.6×10^7^ c.f.u. cm^-2^ (Fig. 1). Bacterial spot is not typically reported on eggplant, but in this case, the diseased plants in the field were surrounded by tomato (Fig. 2).

**Table 1.** Genome statistics of sequenced *Xanthomonas* strains from Turkey as compared to two reference genomes

Strain designation	Host of isolation	Species	No. of contigs	Genome length (Mbp)	Genome coverage (X)	N50 (bp)	Accession no.	Protein coding genes	RNA genes	tRNAs
Tu_01	Tomato	* X. perforans *	37	5.05	102.51	389 719	JAQREE000000000	4220	91	53
Tu_02	Tomato	* X. perforans *	34	5.05	100.56	389 719	JAQRED000000000	4215	91	53
Tu_03	Tomato	* X. perforans *	40	5.05	94.89	368 295	JAQREC000000000	4220	91	53
Tu_04	Eggplant	* X. perforans *	35	4.91	99.05	390 464	JAQREB000000000	4050	89	51
Tu_05	Pepper	* X. euvesicatoria *	147	5.19	91.84	141 309	JAQREA000000000	4312	95	52
Tu_06	Pepper	* X. euvesicatoria *	64	5.05	95.9	210 753	JAQRDZ000000000	4111	95	52
Tu_07	Tomato	* X. perforans *	36	5.05	92.98	368 311	JAQRDY000000000	4216	91	53
Tu_08	Pepper	* X. euvesicatoria *	76	5.15	88.3	197 320	JAQRDX000000000	4239	95	52
Tu_09	Pepper	* X. perforans *	38	5.12	84.3	389 719	JAQRDW000000000	4292	91	53
Tu_10	Pepper	* X. euvesicatoria *	90	5.13	92.29	168 005	JAQRDV000000000	4200	95	52
Tu_11	Pepper	* X. euvesicatoria *	58	5.05	94.26	197 577	JAQRDU000000000	4116	95	52
LMG-27970	Pepper	* X. euvesicatoria *	1491	5.03	72	9186	JPYC00000000	–	–	–
DSM-18975	Tomato	* X. perforans *	3	5.03	435	4 914 073	JABFFS000000000	4138	94	53

A *de novo* assembly of the genomes of 11 *Xanthomonas* strains generated assemblies with the number of contigs ranging from 34 to 147 (average = 60) and sequencing coverage ranging from 84 to 102 (average =94). The genome length varied from 4.91 to 5.18 Mbp with an average of 5.07 Mbp. Genomes had an average *N*_50_ of 291 926 bp. Whole-genome ANI analysis was consistent with initial identifications by real-time PCR. The five *X. euvesicatoria* strains had >99.8 % and 98.5 % ANI with type strain of *X. euvesicatoria* (i.e. LMG_27970) and the type strain of *X. perforans* (i.e. DSM_18975), respectively. The six *X. perforans* strains showed >99.8 % and 98.6 % ANI with the *X. perforans* type strain and *X. euvesicatoria* type strain, respectively. The NCBI annotation for all *X. perforans* strains from Turkey predicted 91 RNA genes and 53 tRNA genes except for Tu_04 strain, which has 89 RNA genes and 51 tRNA genes, whereas all *X. euvesicatoria* strains from Turkey contain 95 RNA genes and 52 tRNA genes. The predicted number of protein coding genes varies from 4050 to 4312 (average =4199) in all 11 strains. Genome statistics of all sequenced strains, NCBI annotation data along with the isolated host are reported in Table 1 and pairwise ANI is presented in Table 2.

**Table 2.** Pairwise average nucleotide identity shown in percentage between the sequenced *Xanthomonas* strains and with type strains. The colour denotes the species identity as; orange= *X. perforans *, blue= *X. euvesicatoria
*

	Tu_01	Tu_02	Tu_03	Tu_04	Tu_05	Tu_06	Tu_07	Tu_08	Tu_09	Tu_10	Tu_11	LMG-27970	DSM-18975
Tu_01	100.00 %	99.99 %	99.99 %	99.82 %	98.49 %	98.52 %	99.99 %	98.52 %	99.99 %	98.52 %	98.51 %	98.55 %	99.91 %
Tu_02	99.99 %	100.00 %	99.99 %	99.81 %	98.49 %	98.53 %	99.99 %	98.53 %	99.99 %	98.52 %	98.51 %	98.55 %	99.91 %
Tu_03	99.99 %	99.99 %	100.00 %	99.82 %	98.49 %	98.53 %	99.99 %	98.53 %	99.99 %	98.52 %	98.51 %	98.56 %	99.91 %
Tu_04	99.82 %	99.82 %	99.82 %	100.00 %	98.57 %	98.57 %	99.82 %	98.60 %	99.81 %	98.59 %	98.56 %	98.61 %	99.84 %
Tu_05	98.57 %	98.59 %	98.54 %	98.63 %	100.00 %	99.94 %	98.57 %	99.97 %	98.56 %	99.92 %	99.94 %	99.88 %	98.56 %
Tu_06	98.58 %	98.59 %	98.56 %	98.62 %	99.92 %	100.00 %	98.57 %	99.95 %	98.57 %	99.91 %	99.98 %	99.88 %	98.58 %
Tu_07	99.99 %	99.99 %	99.99 %	99.81 %	98.49 %	98.53 %	100.00 %	98.52 %	99.99 %	98.52 %	98.51 %	98.55 %	99.91 %
Tu_08	98.57 %	98.59 %	98.54 %	98.63 %	99.94 %	99.95 %	98.57 %	100.00 %	98.56 %	99.89 %	99.95 %	99.89 %	98.56 %
Tu_09	99.99 %	100.00%	99.99 %	99.82 %	98.49 %	98.53 %	99.99 %	98.52 %	100.00 %	98.52 %	98.51 %	98.55 %	99.91 %
Tu_10	98.57 %	98.58 %	98.54 %	98.62 %	99.90 %	99.89 %	98.56 %	99.89 %	98.55 %	100.00 %	99.88 %	99.86 %	98.56 %
Tu_11	98.58 %	98.59 %	98.55 %	98.62 %	99.92 %	99.99 %	98.57 %	99.96 %	98.57 %	99.90 %	100.00 %	99.88 %	98.58 %
LMG-27970	98.56 %	98.59 %	98.57 %	98.63 %	99.83 %	99.86 %	98.56 %	99.86 %	98.56 %	99.85 %	99.87 %	100.00 %	98.55 %
DSM-18975	99.90 %	99.91 %	99.90 %	99.81 %	98.46 %	98.50 %	99.90 %	98.49 %	99.89 %	98.49 %	98.49 %	98.53 %	100.00 %

*X. perforans *, formerly known as bacterial spot pathogen on tomato only, has recently been shown to have expanded its host range to pepper as an emerging pathogen [2, 3, 9, 10]. We found *X. perforans* to be highly pathogenic on pepper and weakly pathogenic on eggplant, supportive of the host expansion. This could have resulted either from gene flow from *Xanthomonas* species infecting pepper [2] or mutations in the genes that affect pathogenicity on pepper[20]. The genetic factors, however, involved in host specificity in *X. perforans* remain poorly understood. Future research investigating the extent of host expansion and adaptation is needed to precisely characterize host–pathogen interaction and understand the associated mechanisms contributing to the successful host expansion of *X. perforans *.
